# Movement smoothness in chronic post-stroke individuals walking in an outdoor environment—A cross-sectional study using IMU sensors

**DOI:** 10.1371/journal.pone.0250100

**Published:** 2021-04-22

**Authors:** Flora do Vale Garcia, Maira Jaqueline da Cunha, Clarissa Pedrini Schuch, Giulia Palermo Schifino, Gustavo Balbinot, Aline Souza Pagnussat

**Affiliations:** 1 Department of Physiotherapy, Universidade Federal de Ciências da Saúde de Porto Alegre (UFCSPA), Porto Alegre, Brazil; 2 Movement Analysis and Rehabilitation Laboratory, UFCSPA, Porto Alegre, Brazil; 3 Rehabilitation Sciences Graduate Program, Universidade Federal de Ciências da Saúde de Porto Alegre (UFCSPA), Porto Alegre, Brazil; 4 KITE-Toronto Rehabilitation Institute, University Health Network, Toronto, Ontario, Canada; 5 Health Sciences Graduate Program, Universidade Federal de Ciências da Saúde de Porto Alegre (UFCSPA), Porto Alegre, Brazil; Baylor College of Medicine, UNITED STATES

## Abstract

**Background:**

Walking speed is often used in the clinic to assess the level of gait impairment following stroke. Nonetheless, post-stroke individuals may employ the same walking speed but at a distinct movement quality. The main objective of this study was to explore a novel movement quality metric, the estimation of gait smoothness by the spectral arc length (SPARC), in individuals with a chronic stroke displaying mild/moderate or severe motor impairment while walking in an outdoor environment. Also, to quantify the correlation between SPARC, gait speed, motor impairment, and lower limb spasticity focused on understanding the relationship between the movement smoothness metric and common clinical assessments.

**Methods:**

Thirty-two individuals with a chronic stroke and 32 control subjects participated in this study. The 10 meters walking test (10 MWT) was performed at the self-selected speed in an outdoor environment. The 10 MWT was instrumented with an inertial measurement unit system (IMU), which afforded the extraction of trunk angular velocities (yaw, roll, and pitch) and subsequent SPARC calculation.

**Results:**

Movement smoothness was not influenced by gait speed in the control group, indicating that SPARC may constitute an additional and independent metric in the gait assessment. Individuals with a chronic stroke displayed reduced smoothness in the yaw and roll angular velocities (lower SPARC) compared with the control group. Also, severely impaired participants presented greater variability in smoothness along the 10 MWT. In the stroke group, a smoother gait in the pitch angular velocity was correlated with lower limb spasticity, likely indicating adaptive use of spasticity to maintain the pendular walking mechanics. Conversely, reduced smoothness in the roll angular velocity was related to pronounced spasticity.

**Conclusions:**

Individuals with a chronic stroke displayed reduced smoothness in the yaw and roll angular velocities while walking in an outdoor environment. The quantification of gait smoothness using the SPARC metric may represent an additional outcome in clinical assessments of gait in individuals with a chronic stroke.

## Introduction

Individuals with chronic stroke often display reduced motor control, muscle weakness, and spasticity in the lower limbs [1,2]. This may lead to asymmetries while standing or walking [3], slower gait with poor coordination [4], greater metabolic consumption while walking [5], and postural instability, ultimately leading to reduced community ambulation [6]. In the clinic, gait assessments often include the quantification of gait speed, which is easily and reliably measured using a chronometer and a fixed distance. This assessment is considered a good indicator of the overall walking performance in individuals with stroke and may be accomplished, for example, using the 10 meters walking test (10 MWT) [7–9]. Although gait speed and functional scales provide a broad picture of the post-stroke gait impairment, the detailed assessments of postural behavior and instability are usually accomplished using kinematics or kinetics. Similar to gait speed and functional scales, the gait spatiotemporal symmetry represents a good metric to estimate the above-mentioned walking asymmetries and is associated with gait speed in the stroke gait [3]. However, gait speed and spatiotemporal symmetry offer limited information about the deficits in dynamic posture and smoothness during walking, including the compensatory postural adjustments to maintain/change symmetry and speed [10,11]. Thus, movement smoothness estimated from a waist-mounted inertial measurement unit (IMU) may constitute an important additional metric to quantifying the quality of movement [10,11], including postural adjustments—since one may walk with a perfectly symmetric but unsmooth gait pattern [12].

An adequate movement smoothness has been associated with satisfactory scores of balance [13,14], motor control [15], and gait speed [10]. Recently, the spectral arc length (SPARC) was proposed to quantify point-to-point reach in stroke, with excellent ability to deal with duration artifacts. Longer movements may overshadow other movement smoothness metrics such as trajectory, velocity, and acceleration (jerk) adjustments. SPARC measurement assumes less smooth movements are more complex (more frequency composition) when approaching the original signal with a Fourier series. Therefore, it measures the movement smoothness by defining the signal’s complexity by the power spectrum profile [16]. In point-to-point reach tasks, the use of SPARC was shown to outstand traditional and adjusted smoothness measures [12]. Importantly, SPARC was recently adapted and recommended for estimating movement smoothness using data extracted from IMUs [17]. Thus, SPARC constitutes an optimal measure of smoothness given the reduced impact of duration—since the analysis is conducted in the frequency-domain. Evaluation of movement smoothness is useful in neurorehabilitation because it may enable the quantification of motor recovery [18,19] and predict post-stroke motor control [14,15] in great detail. Also, as mentioned earlier, the ability to account for the postural adjustment and compensations may provide important information, especially in the cases of post-stroke individuals with fast and symmetric gait patterns.

Quantifying acceleration, deceleration, and angular velocity using a lumbar IMU affords the estimation of the intermittency of rhythmic movements. The presence of such intermittencies in movement is quantified by the SPARC metric, which reflects the presence of movement arrest periods [17,18,20]. IMUs are noninvasive inertial sensors used to assess gait in the laboratory and outdoor environments [12,20,21]. The portability of IMU systems allows the investigation of gait biomechanics outside the laboratory environment. Walking in outdoor environments may be challenging for individuals with motor impairment, especially those with severe compromise [22,23]. For this reason, it is interesting to evaluate individuals with stroke and with different levels of motor impairment while walking in "real contexts" outside the laboratory setting. Natural constraints of the environment may provide a better picture of the ’real-life’ gait impairments that stroke survivors face in daily walking activities. Thereby, we think it may provide a valid approach to assess the presence of movement arrest periods in stroke individuals whose motor impairment was previously classified in the laboratory environment. IMUs are a good alternative to evaluate such movement arrest periods in real contexts and mitigate typical limitations of laboratory settings–such as the highly controlled environment and reduced experimental area [24].

Several metrics are described in the literature to quantify smoothness [20], such as the jerk [11] and the harmonic ratio [12]. However, SPARC has been pointed out as a superior approach compared to others [11,25]. SPARC captures the movement intermittency, e.g., movement arrest periods [17], with reduced influence of movement amplitude or duration, and has been used to quantify gait smoothness in the oldest-old [26] and individuals with Parkinson’s disease [11,21]. Nonetheless, no study to date explored the ability of SPARC obtained from IMU sensors in quantifying the hemiplegic gait. Considering the use of gait smoothness as a complement to the traditional gait speed assessment, this study aimed: a) to quantify gait smoothness (SPARC) in individuals with a chronic stroke while walking in an outdoor environment; b) to compare SPARC features of post-stroke individuals with mild/moderate and severe motor impairment; and c) to explore the correlation of SPARC with gait speed, gait symmetry, motor impairment, and lower limb spasticity. We hypothesized that SPARC would quantify the gait deficits in individuals with a stroke, represent an additional metric to complement the traditional gait speed assessment, and correlate with clinical assessments of motor impairment and spasticity.

## Materials and methods

### Study design

This cross-sectional study was approved by the Ethics and Research Committee of the Santa Casa de Misericórdia Hospital of Porto Alegre (CAAE 64819617.0.0000.5335) and was conducted according to the Strengthening the Reporting of Observational Studies in Epidemiology (STROBE) checklist [27].

### Participants

Volunteers were recruited through a Santa Casa de Misericórdia Neurology service database in Porto Alegre, Brazil, institutional sites, and social media. Those individuals who met the following criteria were included as convenience sample: aged between 18 and 80 years; with a diagnosis of cortical or subcortical unilateral cerebrovascular accident confirmed by imaging (tomography or magnetic resonance imaging); time since the stroke from 6 months to 10 years; ability to walk at least 10 meters with or without assistive devices; minimum score of 20/30 points (illiterate) or > 24/30 points (literate) in the Mini-Mental State Examination (MMSE) [28]. Individuals with a clinical diagnosis of musculoskeletal diseases, significant visual deficit, and history of falls in the last 3 months were excluded.

The study also included a reference group of healthy individuals, paired by age and sex. Exclusion criteria for the reference group were: previous history of neurological or musculoskeletal disorders that induced visible gait abnormalities. All participants signed the informed consent after an explanation about the objectives and possible complications of the study. According to the MMSE score, all participants were presumed competent for decision-making and, for this reason, signed the informed consent (Table 1).

**Table 1:** Demographic characteristics.

	Control (n = 32)	Stroke (n = 32)	*p = value*	Mild/Moderate impairment (n = 16)	Severe impairment (n = 16)	*p = value*
**Gender, n (%)**						
**Male**	22 (68.8)	22 (68.8)	1.000	10 (62.5)	12 (75)	0.444
**Age, years, mean ±SD**	56.81 ± 8.88	56.84 ± 9.10	0.989	55.88 ± 11.42	57.81 ± 6.22	0.557
**Height (m)**	1.72 ± 8.23	1.68 ± 9.73	0.221	168.63 ± 9.74	170.56 ± 7.86	0.540
**Body mass (kg)**	78.26 ± 12.97	75.13 ±11.90	0.318	76.13 ± 12.27	74.13 ± 11.83	0.642
**MMSE Score, median (min-max)**	30 (27–30)	29 (25–30)	<0.001*			
**Time since stroke, mth, median (min-max)**		39.41 (6–96)		43 (6–96)	23 (6–84)	0.220
**Stroke type, n (%)**						
**Ischemic**		24 (75)		11(68.75)	13 (81.25)	0.412
**Hemorrhagic**		8 (25)		5 (31.25)	3 (18.75)	
**Affected hemisphere, n (%)**						
**Right**		19 (59.4)		9 (56.2)	10 (62.5)	0.719
**Left**		13 (40.6)		7 (43.8)	6 (37.5)	
**FMA-LL (0–34), median (min-max)**		19.63 (11–32)		23 (20–32)	15 (11–18)	<0.001*
**MAS, frequency (0/1/1^+^/2/3/4)**						
**Plantiflexors**	0/3/2/4/11/12			0/2/2/4/4/4	0/1/0/0/7/8	0.029*
**Knee extensors**	5/7/6/4/8/2			4/5/2/2/3/0	1/2/4/2/5/2	0.033*
**Adductors**	5/4/4/12/7/0			4/3/3/4/2/0	1/1/1/8/5/0	0.021*

*FMA-LL*, *Fugl Meyer Assessment—Lower Limb; MAS*, *Modified Ashworth Scale; max*, *maximum; min*, *minimum; n*, *number of participants; SD*, *standard deviation*. Mild-Moderate: FMA-LL scores of 20–34; Severe: FMA-LL scores below than 19. t-Student and U-Mann-Whitney tests were used for parametric or nonparametric data, respectively. Likelihood Ratio Chi-Square Tests were used for data of gender, type of stroke, affected hemisphere and MAS.

### Procedures

This study was conducted at the Federal University of Health Sciences of Porto Alegre (UFCSPA) between January 2018 and May 2019. Each participant performed a clinical and documented evaluation session. The same researcher applied all clinical assessments.

### Clinical measurement

#### Lower limb motor impairment

The motor function domain of Fugl-Meyer Assessment (FMA) was used to assess lower limb (LL) motor impairment [29]. We included only the motor domain scale, without other domains (nonmotor domains: sensation, balance, joint range of motion, and joint pain). The motor subscale measures the voluntary movement, velocity, coordination, and reflex activity about the hip, knee, and ankle. Each item has three possible scores, 0 (cannot be performed), 1 (partially performed), and 2 (performed entirely). The maximum score for the LL is 34 points. According to the FMA-LL score, the participants were classified following their motor impairment as severe (0 to 19), moderate (20–28), or mild (≥29 points) [30].

#### Modified Ashworth Scale (MAS)

MAS was used to evaluate resistance to passive movements. This measure corresponds to an indirect assessment of spasticity [31]. This scale consists of 6 ordinal values ranging from 0 (no tonus increase) to 4 (stiffness) [32]. Participants were evaluated lying in the supine position and instructed to remain relaxed during the test. Spasticity of plantar flexors, knee extensors, and hip adductors was tested.

#### Gait assessment

**Fig 1:**
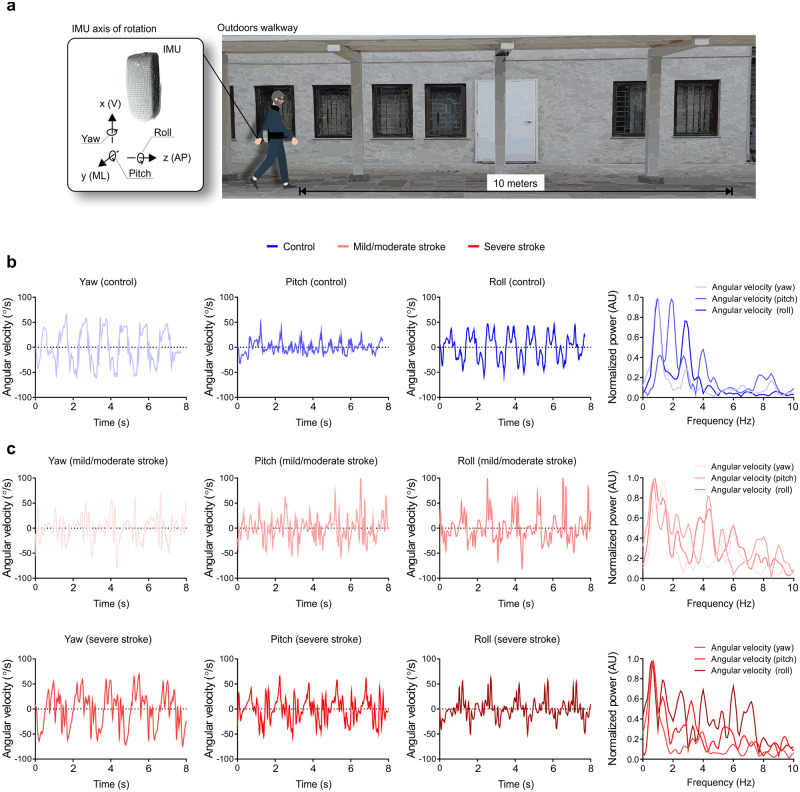
Experimental setup. (**a**) The instrumented 10 meters walking test (10 MWT) was performed outdoors using an inertial measurement unit (IMU) attached to the participant’s waist and connected to a laptop (via Bluetooth). (**b**) Representative yaw, pitch, and roll angular velocities of the control group (blue lines). **(c, d)** Representative yaw, pitch, and roll angular velocities of stroke participants with mild/moderate (light red lines) and severe (dark red lines) impairment. Data were analyzed using time series of a non-overlapping moving window of 3 seconds. The normalized power (right panels) represents the last analyzed window for each of the angular velocities.

Participants were requested to walk at a self-selected speed on a 10-meters flat pathway of the university outdoors. This pathway was sheltered from the rain, but participants regularly crossed with other people and received several sensory stimuli during the task. Data were acquired at least twice for each participant. During the evaluation, participants wore an inertial sensor (BTS G-Walk BTS Bioengineering Corporation, Italy, with a sampling rate of 100 Hz) that measured the acceleration along three accelerations axes: vertical acceleration (V), mediolateral acceleration (ML), anterior posterior acceleration (AP); and three angular velocity axes: yaw, pitch, and roll. In the smoothness analysis, we only consider the angular velocity axes. The inertial sensor was attached to the subjects’ waists with a semi-elastic belt, covering the L5 S1 segments [33]. Acceleration data were transmitted via Bluetooth to a PC and processed using dedicated software (BTS G-STUDIO, version: 2.6.12.0) (Fig 1). Finally, offline processing of the acquired acceleration signals was performed as described below.

### Data processing

Data was calculated over an average of 2–3 trials for controls, 2–5 trials for mild stroke, and 2–3 trials for severe stroke (≈ 32–40 steps to 48–60 strides or 96–120 steps). To determine the onset and offset of movement, the researcher was visually guided by the pitch angle, which depicts the anteroposterior body oscillations. The onset was defined when the pitch angle reached its first peak, corresponding to the first step, and the offset when the pitch angle presented the last peak, corresponding to the last step. Portions of the signal outside these limits were excluded and deemed to contain acceleration and deceleration phases of the gait, respectively.

#### Spectral arc length (SPARC)

Raw data from the G-Studio software was extracted, exported as ASCII files, and analyzed offline using LabVIEW^®^ (National Instruments, USA, v.18.0) custom software routines to obtain speed and smoothness variables.

Gait smoothness was estimated using the SPARC metrics, as Beck et al., and Pinto et. al. [11,21] described previously. The algorithm was further optimized following the recent recommendations for estimating SPARC from IMUs [17]. We used the 3 calibrated angular velocities (yaw, roll, and pitch) (in °/s) from the IMU gyroscope data, mean subtractions were used to remove the direct current (DC) components from raw angular velocity data, and whenever signal manipulations caused the drifting of the signal. Since SPARC was initially developed for discrete point-to-point reaching, we also implemented a windowing procedure to reduce the effects of long, continuous data windows–often present during the 10MWT in stroke participants. Very long windows may increase the FFT resolution and the frequency complexity, respectively, which can induce a duration effect over SPARC. A conservative non-overlapping window size of 3 seconds (300 frames) was used to represent at least 1 full stride cycle in pathological gait. If the last window was less than 3 seconds, this window was automatically excluded by the algorithm. Each cropped window was zero-padded to double the FFT frequency resolution, and SPARC was calculated. For SPARC calculation, the high frequencies not involved in pathological gait were removed when applying the limits of integration (lower bound = 0; upper bound = 10 Hz) [12]. The upper bound of 10 (ω_c_: adaptive cut-off frequency) was chosen based on the presence of higher frequencies in pathological gaits, such as Parkinson’s [21].

We calculated the SPARC $\left[\lambda_{S}^{v}\left(V\right)\right]$ for each 3 seconds window. The average of all SPARC windows contained in the walking trial was calculated.

$$\lambda_{S}^{v}\left(V\right)≜-\int_{0}^{\omega_{c}}\sqrt{\left[{\left(\frac{1}{\omega_{c}}\right)}^{2}+{\left(\frac{d\hat{V}\left(\omega \right)}{d\omega }\right)}^{2}\right]}d\omega$$

$$\hat{V}\left(\omega \right)=\frac{V\left(\omega \right)}{V\left(0\right)};\;V(\omega )=\left|F({\left\|V\left(t\right)\right\|}_{2})\right|$$ (1)

*where V (t) represents the velocity (angular) of a movement in the time domain*, *F(•) is the Fourier transform operator*, *ωc is an adaptive cut-off frequency (see Melendez-Calderon et al*., *2021* [17] *for details)*.

Movement arrest periods increase the complexity of the frequency composition, which is captured by the SPARC analysis. Because of the negative sign in (1), lower SPARC values indicate less movement smoothness (increased frequency domain complexity).

Also, we conducted a short-time Fourier transform (STFT)-based spectrogram of the angular velocity signals to show representative spectrograms of walking trials. For this, we applied 512 frequency bins resulting in 256 frequency scales with a resolution of 0.19 Hz using the TFA STFT Spectrogram VI in LabVIEW.

Spectral analysis metrics followed the assumption that unsmooth movements are more complex in terms of their frequency composition. In other words, lower SPARC values indicate less movement smoothness [16].

*Acceleration adjustments (Jerk)*. The angular velocity time-series was filtered using a low-pass 1^st^ order Butterworth filter at 10 Hz. The filtered angular velocities were derivated to achieve the angular acceleration. The angular acceleration profile was derived to obtain the acceleration variation (jerk). The initial and final frames were removed after each derivation to remove artifacts. To quantify jerk, we counted the number of zero-crossings in the jerk profile.

*Gait symmetry and speed*. The typical spatiotemporal gait parameters are automatically obtained using the dedicated BTS G-STUDIO software from the acceleration signals. Gait velocity was computed as the mean speed by the ratio of walking distance to duration. Step length was estimated based on the amplitude of vertical pelvic displacement and leg length using a simple inverted pendulum model of walking. It was calculated based on the approach described by the literature [33,34]. The gait symmetry (%) was quantified according to the method described by Robinson [35] as follows:

$$SI=\left[\frac{2(x\;non\;paretic-x\;paretic)}{\left(x\;non\;paretic+x\;paretic\right)}\right]x100$$ (2)

*where SI is symmetry index in step lengths; x non-paretic is* the value of the variable obtained from the non-paretic limb; *x paretic is* the value of the corresponding variable obtained from the paretic limb.

The symmetry index was calculated by the quotient of the difference between step length for paretic and non-paretic limb. The symmetry index should be zero, reflecting a perfectly symmetrical gait pattern. Higher symmetry index values correspond to greater gait asymmetry [35,36].

### Statistical analysis

The sample size was determined by G-Power 3.0 software based on previous studies [11,21] adopting 90% power and the alpha value of 0.05 to detect the minimum effect size of 0.7% in SPARC [21]. A total of 30 participants in each group (stroke and control) was estimated as necessary to this study.

Data were expressed as mean, standard deviation (SD) or 95% confidence intervals (continuous variables) and frequency distribution (categorical variables). Shapiro-Wilk tests were used to evaluate the normality of the continuous variables. Parametric Student t-tests, nonparametric Mann–Whitney, and Chi-square tests were used to compare demographics and stroke-related characteristics between groups. To classify the level of impairment in the stroke group the FMA-LL scores were used: mild/moderate of 20–34 and severe below 19 points. One way-ANCOVA was conducted to compare SPARC and symmetry across three groups (mild/moderate stroke, severe stroke, and control) while controlling for gait speed. Levene’s test and normality checks were carried out. One way-ANOVA was used to compare the SD of SPARC throughout the analysis windows. Tukey posthoc test was applied when necessary. The analysis of variation between windows three control subjects was excluded since they had only 1 valid window for analysis (duration < 6 seconds).

A correlational analysis was conducted between the smoothness variables and gait speed, Symmetry index, FMA-LL, and spasticity using the Person’s and Spearman’s correlation coefficients were used for parametric and nonparametric variables, respectively. We interpreted the strength of correlation as follows: 0.26–0.49 = weak; 0.5–0.69 = moderate; 0.7–0.89 = strong; and 0.9–1.0 = very strong. We used the SPSS statistical software version 21.0 for all analyses. The significance levels were set at p < 0.05.

## Results

Forty-one individuals with chronic stroke were recruited. Nine participants failed to meet all the eligibility criteria and were excluded from the analysis: incapacity to walk 10 meters (n = 3), musculoskeletal disorders (n = 3), cognitive impairment (n = 2), and significant visual deficit (n = 1). Thus, thirty-two individuals with chronic stroke and thirty-two healthy controls were included. According to the impairment level, post-stroke individuals were divided into two groups: Mild/Moderate (n = 16, 10 men, 55.88 ± 11.42 years), and Severe (n = 16, 12 men; 58.81 ± 6.22 years). The demographic data and clinical characteristics of participants are presented in Table 1.

Stroke individuals presented lower gait speed with higher asymmetry compared with controls. Gait symmetry (%) was of 2.82 (1.55–4.85) [median and 25^th^ to 75^th^ inter-quartile range] for controls, 12.03 (6.43–29.6) for mild/moderate and 7.75 (3.81–27.44) for severe individuals with severe compromise (U_(2)_ = 23.21; p<0.001). Subgroup analysis showed no differences between mild/moderate and severe stroke participants (p > 0.05). Gait speed was of 1.22 ± 0.17 m.s^-1^ for controls, 0.68 ± 0.24 m.s^-1^ for mild/moderate and 0.58 ± 0.27 m.s^-1^ for individuals with severe compromise (F_(2,61)_ = 60.18, p < 0.001). Posthoc indicated the control group was faster than both mild/moderate and severe stroke groups (p < 0.05). Given the strong influence of velocity over the groups, we decided to account for gait speed as a covariate in our group analysis.

**Fig 2:**
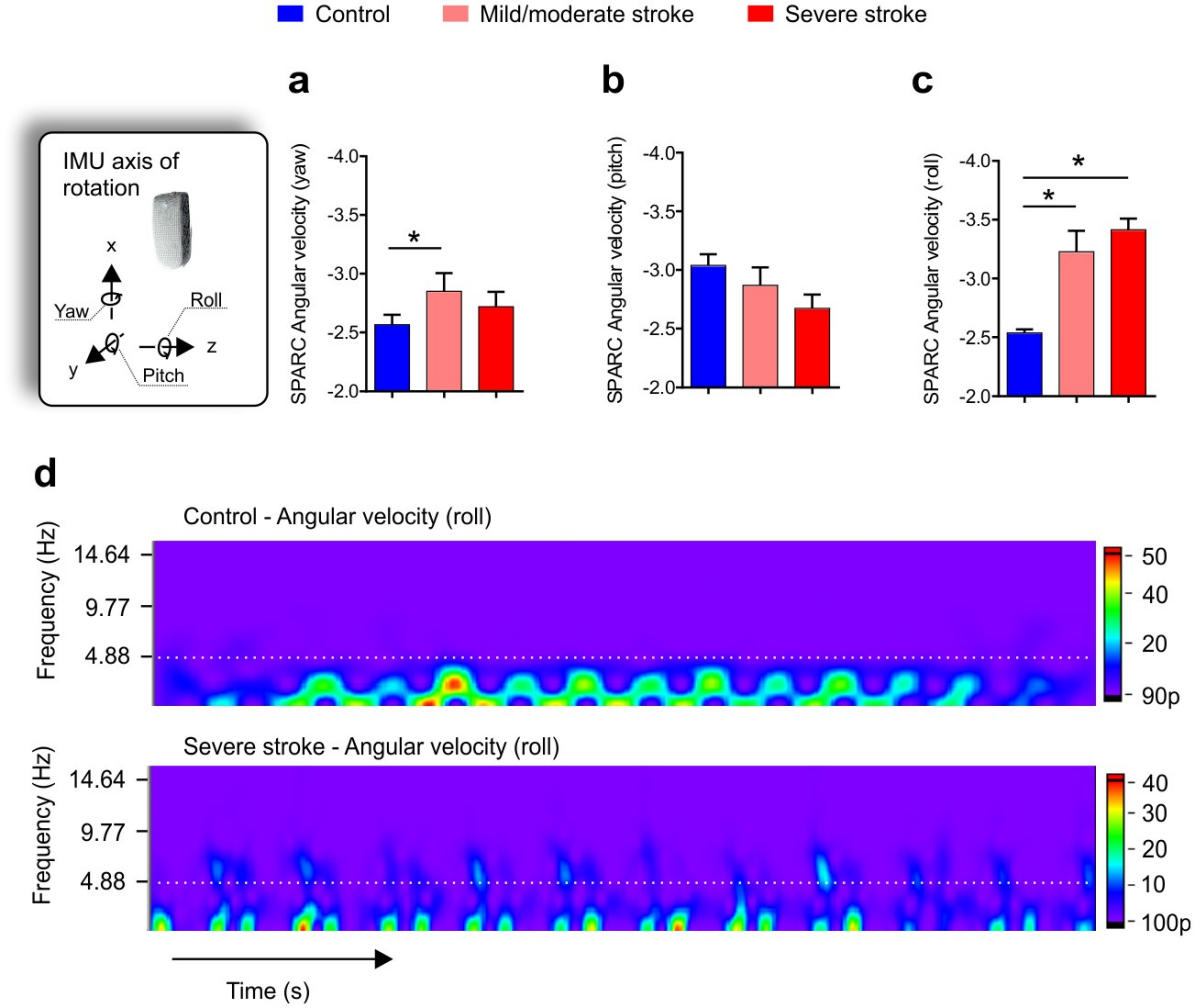
SPARC angular velocities profiles and representative spectral-domain of healthy control and severely impaired stroke participants during the outdoor 10-meters walk test (10 MWT). **(a,b,c)** Average of yaw, pitch, and roll angular velocities for the control group (blue) and stroke participants with mild/moderate impairment (light red) and severe impairment (dark red). Overall, stroke participants presented greater roll angular velocity than the control group (p <0.001). **(d)** Representative heatmap of power spectral density for roll angular velocity. Note the severely impaired stroke participants presented frequency magnitude peaks above the 5Hz compared with healthy controls (white hashed lines). One-way ANCOVA with Bonferroni posthoc correction, *p < 0.05.

A one-way ANCOVA revealed a significant difference in mean SPARC angular velocity (yaw) (F_(2,60)_ = 4.317, p = 0.018) between the groups while adjusting for gait speed. Post-hoc comparisons showed that the mild/moderate (p = 0.015) stroke group presented less smooth gait than the control group (Fig 2a). For the SPARC angular velocity (pitch) there was no significant difference between groups accounting for gait speed as covariate (F_(2,60)_ = 1.888, p = 0.160, Fig 2b). There was a significant difference in mean SPARC angular velocity (roll) between the groups (F_(2,60)_ = 4.413, p = 0.016). Post-hoc comparisons revealed that mild/moderate (p = 0.024) and severe (p = 0.021) stroke groups presented less smooth gait than the control group (Fig 2c). Overall, the severely impaired stroke participants presented greater intensity in higher frequencies (i.e., > 5 Hz) compared to controls, as shown in Fig 2d.

**Fig 3:**
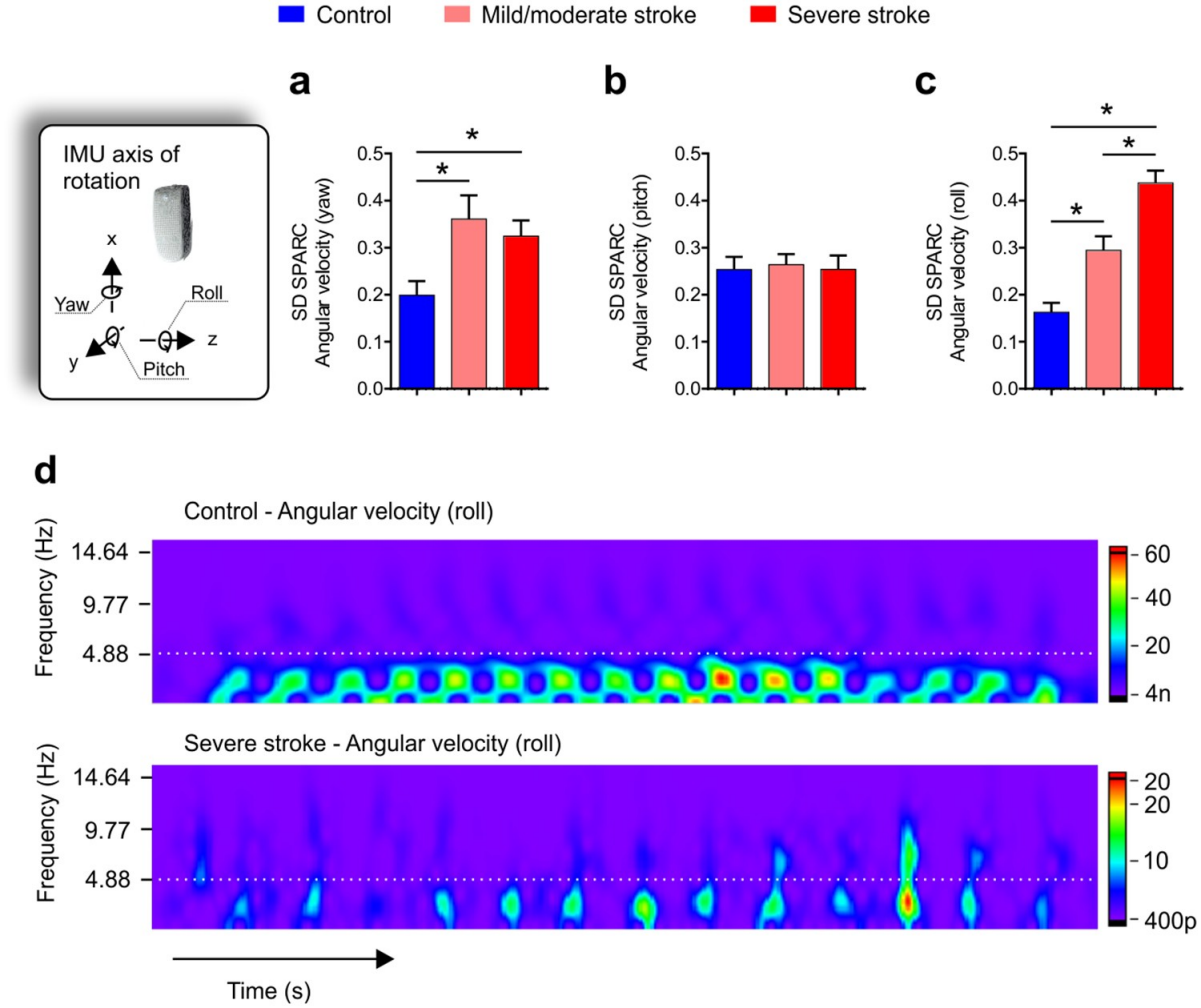
Variability of SPARC angular velocities profiles and representative spectral-domain of the control group and severely impaired stroke participants during the outdoor 10-meters walk test (10 MWT). **(a,b,c)** Standard deviation (SD) throughout the 3-second analysis windows of yaw, pitch, and roll angular velocities for the control group (blue) and stroke participants with mild/moderate impairment (light red) and severe impairment (dark red). Overall, stroke participants presented greater variability of smoothness during the walking trial for yaw and roll angular velocities than controls (p < 0.05). **(d)** Representative heatmap of power spectral density for roll angular velocity. Note the severely impaired stroke participants presented frequency magnitude peaks above the 5Hz compared with controls (white hashed lines). Also, note that the severely impaired stroke participants presented more variable frequency magnitude peaks across the 10 MWT than the control group. One way ANOVA with Tukey’s posthoc correction, *p < 0.05.

When analyzing the variation (SD) of smoothness along the 10 MWT across all the 3-seconds analysis windows, a one-way ANOVA indicated stroke participants presented more variation in smoothness during the walking trial. For SD SPARC angular velocity (pitch), there was a group effect (F_(2,59)_ = 5.931, p = 0.0028, Fig 3a), in which both mild/moderate and severe stroke groups showed greater variability of smoothness during the 10 MWT (p < 0.05). No differences were evident for SPARC variability in the pitch angular velocities [SD SPARC angular velocity (pitch); Fig 3b]. Roll angular velocity smoothness also showed a group effect (F_(2,59)_ = 33.56, p < 0.0001, Fig 3c). This result indicated both stroke groups were more variable in terms of smoothness than controls, but the severe stroke group was more variable than the mild/moderate group. Note the higher frequency amplitude is less constant for the severe stroke group, showing a remarkable high intensity at the end of the walking trial (Fig 3d).

Overall, unlike healthy controls, post-stroke individuals showed a broad spectral range that encompassed several dominant frequencies, not restricted to lower frequencies (Figs 1c, 1d, 2d and 3d). Control individuals displayed lower frequency components, indicating low variability of events generated by stride and step frequencies during walking. A greater number of well-defined higher frequencies in post-stroke individuals was evident—likely reflecting the distinct strategies employed during walking.

**Fig 4:**
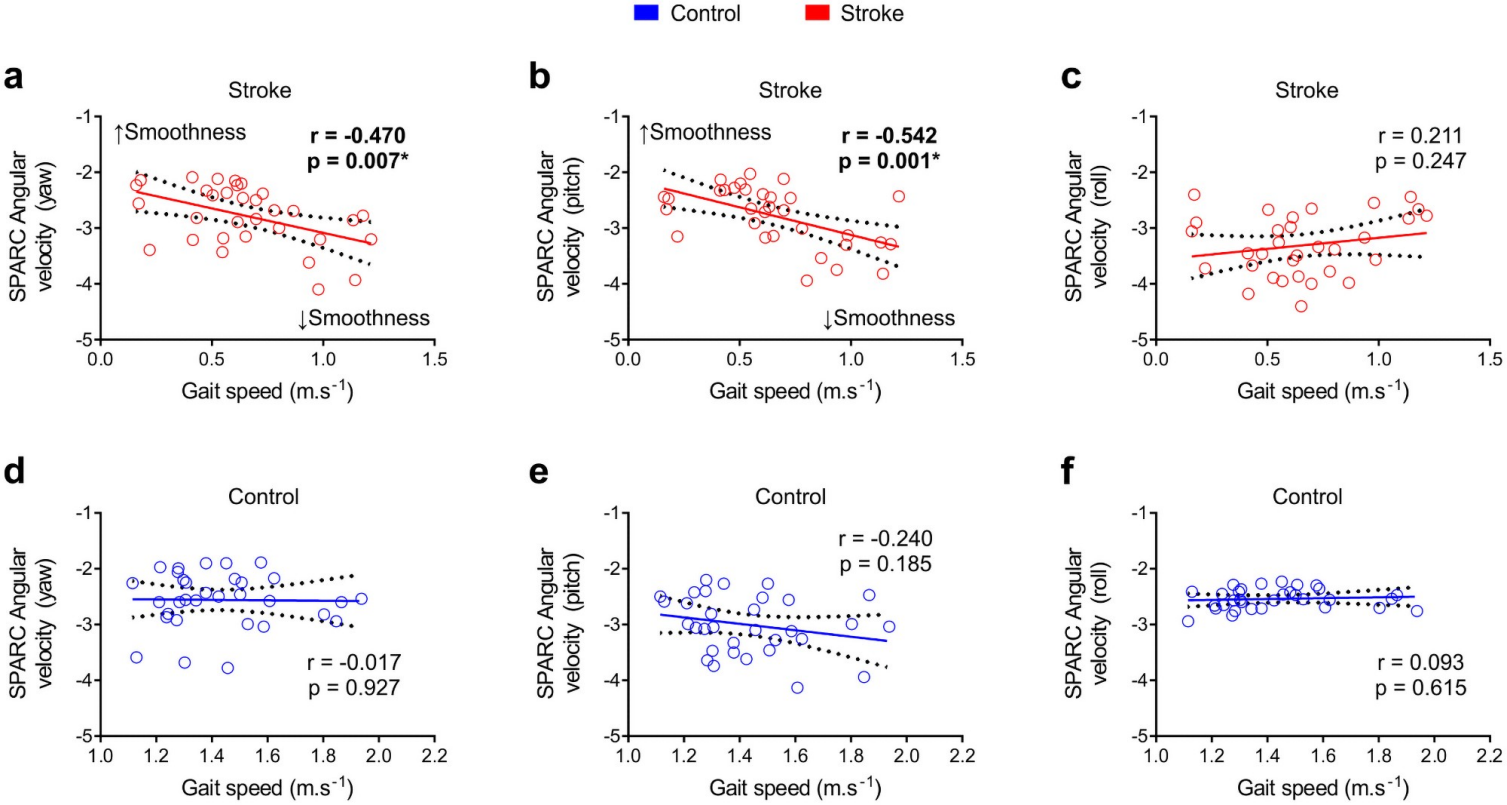
Correlations between SPARC and gait speed for the stroke group indicated faster speeds were associated with reduced smoothness. (**a**) There was a significant correlation between SPARC angular velocity (yaw) and gait velocity in the stroke group. (**b**) This correlation was also significant between SPARC angular velocity (pitch) and gait velocity in the stroke group. (**c**) The correlation between SPARC angular velocity (roll) and gait velocity in the stroke group was not significant. (**d-f**) Correlations between SPARC angular velocity (yaw, pitch, and roll) and gait velocity were not significant in the control group. The dashed line represents the 95% confidence interval; Pearson correlation, *p < 0.05.

Table 2 shows correlations between gait speed and asymmetry, clinical variables (FMA-LL and MAS), and SPARC. We found SPARC angular velocity (yaw and pitch) presented a moderate correlation with gait speed. These correlations indicated a negative relation between SPARC and gait speed, where a reduced smoothness was related to faster speeds (Fig 4a and 4b), not evident in controls (Fig 4d and 4e). SPARC angular velocity (roll) was not related to gait speed in both groups (Fig 4c and 4f). FMA-LL and symmetry index were not related to gait smoothness. On the other hand, SPARC angular velocity pitch and yaw displayed a positive and weak/moderate correlation with spasticity of plantarflexors and hip adductors. Also, knee extensors showed a weak negative correlation with SPARC in the roll angular velocity.

**Table 2:** Correlations between clinical parameters and SPARC values in the stroke and control groups.

		Stroke SPARC Angular velocity (yaw)	Stroke SPARC Angular velocity (pitch)	Stroke SPARC Angular velocity (roll)	Control SPARC Angular velocity (yaw)	Control SPARC Angular velocity (pitch)	Control SPARC Angular velocity (roll)
**Gait Speed** ^**#**^	R	**-0.47**	**-0.542**	0.211	-0.017	-0.24	0.093
	*p-value*	0.007*	0.001*	0.247	0.927	0.185	0.615
	n	**32**	**32**	32	32	32	32
**Gait Symmetry Step Length** ^**¥**^	R	0.067	0.264	-0.283	0.065	-0.007	-0.287
	*p-value*	0.714	0.144	0.199	0.723	0.969	0.111
	n	32	32	32	32	32	32
**FMA_LL** ^**#**^	R	-0.333	-0.278	0.161			
	*p-value*	0.067	0.124	0.377			
	n	32	32	32			
**Spasticity, MAS** ^**¥**^							
**Plantiflexors**	R	0.313	**0.505**	-0.248			
	*p-value*	0.081	0.003*	0.171			
	n	32	**32**	32			
**Knee Extensors**	R	0.275	**0.324**	**-0.46**			
	*p-value*	0.128	0.002*	0.002*			
	n	32	**32**	**32**			
**Adductors**	R	**0.385**	**0.437**	-0.321			
	*p-value*	0.029*	0.012*	0.073			
	n	**32**	**32**	32			

Note: FMA-LL, Fugl Meyer Assessment–Lower Limb; MAS, Modified Ashworth Scale; n = number of participants; Pearson’s correlations (p < 0.05).

## Discussion

Individuals with a chronic stroke displayed reduced smoothness while walking in an outdoor environment, indicating movement arrest periods in the yaw and roll angular velocities, and higher spatiotemporal asymmetry compared with control individuals. Severely impaired individuals presented pronounced gait smoothness variability along the walking trajectory, and this greater and variable pattern occurred in the mediolateral roll and yaw angular velocities. These findings are likely associated with mediolateral trunk instability, which is a characteristic of the stroke gait. Gait smoothness quantified using the SPARC metrics also showed independence of speed in the control group, which indicates SPARC is a good complementary metric to the traditional gait speed assessment in the clinic. Our results also demonstrated that pitch smoothness, which reflects the step-to-step pendular mechanics in the sagittal plane, was positively correlated with plantarflexors, knee extensors, and hip adductors spasticity. It was suggestive of the adaptive use of spasticity in maintaining efficient walking. Conversely, knee extensor spasticity was negatively correlated with smoothness of the roll angular velocity, thus, contributing to the evident gait smoothness deficit in this component. We suggest that gait smoothness extracted from IMUs may represent a complementary outcome measure to the traditional gait speed assessments, often employed in the clinic.

A slower gait in individuals with stroke was expected, considering that stroke affects walking speed [37]. Poor motor control, reduced intermuscular coordination [38], and gait instability [39] may decrease velocity and smoothness during walking. The severity of the stroke lesion influences gait speed; nonetheless, some patients with mild motor paralysis may still walk slowly [40]. In this line of thought, the movement quality metric presented here may provide additional gait quality indicators in these specific patients with slower gait but mild motor symptoms. Our results indicated that smoothness in the roll angular velocity showed the ability to capture the post-stroke impairment and was not affected by gait speed. As such, it may circumvent these above-mentioned gait speed influences. Individuals with mild motor paralysis and slow walking speed may display reduced trunk stability [40]. The IMU placement used in the present study (waist-mounted) may, therefore, present important advantages in capturing these subtle trunk oscillations. Thus, SPARC is a good metric in assessing gait smoothness because it is less dependent on the duration of the gait, for example, compared to the Jerk metric, which is more susceptible to duration effects (see S1 Table).

Previous research suggests that gains in gait speed are related to improvements in motor control [41]. Gait speed is the leading indicator of function, level of disability, survival and is broadly used to classify ambulation status after stroke [9,42]. In upper limb studies of reach to grasp movements, moving faster can increase rates of muscle activation that controls proximal and distal multi-joint movements [43]. For example, the reach paths are straighter, finger movements are more efficient, and the fingers open wider [43]. Similarly, point-to-point reach movements recover first by regaining the ability to generate submovements, followed by reacquiring the means to combine submovements [44,45]. This impacts upper limb movement smoothness because the submovements become fewer, longer, and faster with recovery [45]. It is thought that, overall, it is hard for humans to execute slow upper limb movements with smoothness and rhythmicity [46]. Here, individuals with a stroke displayed slower gait speed and reduced smoothness than the reference group, corroborating the findings from upper limb studies. Nonetheless, it is essential to highlight the differences between upper and lower limb studies on movement smoothness. Conversely to reach-to-grasp movements, gait involves full body weight support and greater walking speeds are achieved by providing body weight [47]. Dealing with gravity seems to be a major difference between upper and lower limb studies on movement smoothness. It is straightforward to think of a faster and smoother reach-to-grasp trajectory if performed in a straight line, which would be the shortest path in a point-to-point reach task.

On the other hand, gait is a cyclic process consisting of several stride cycles used to travel to the endpoint (e.g., here the 10 MWT mark), therefore, should rather be considered in terms of step-to-step transitions. Walking speed would affect the stride cycle submovements, such as stance, double-support, and single support phases. In a sub-acute stroke, walking faster may improve spatiotemporal symmetry in double- and single-support proportions between paretic and nonparetic sides [47]. For example, a greater proportion of single support using the paretic limb was evident when walking faster, suggesting increased usage of this limb during the gait cycle [47]. Several studies assessed movement smoothness in individuals with stroke using a waist-mounted accelerometer [48–52], and pieces of evidence on the effect of speed are lacking. It is known that, in older adults, greater walking speeds may induce less smooth accelerations in ML and AP directions [53]. Here, we suggest that greater spatiotemporal symmetry at faster speeds may be afforded by greater dependence on the paretic lower limb with respectively reduced smoothness.

Smoothness was also correlated with the adaptive use of spasticity in individuals with stroke. We found that greater lower limb spasticity was related to gait smoothness in the pitch component. Prior studies conducted in the upper limbs have demonstrated that increased spasticity affects inter-joint coordination during reaching tasks, causing a reduction in movement smoothness [54–56]. As mentioned earlier, movement intermittency in gait must be considered in terms of step-to-step transitions, conversely to upper limb studies that focus on point-to-point movements. Movements in the pitch direction reflect the body pendular motion in the sagittal plane, where the lower limbs support step-to-step transitions efficiently [57]. In post-stroke individuals, the lower limb is thought to act as a rigid shaft supporting the pendular transduction during walking, which is maintained to some extent despite the motor compromise [5]. We suggest that individuals sustaining a chronic stroke learn to adapt the walking dynamics to maintain an efficient gait. They can maintain a smooth angular velocity profile in the pitch angle, which is likely supported by the adaptive use of spastic muscles. Conversely, spasticity was detrimental to maintaining smoothness in the roll angular velocity and support the importance of the roll component of SPARC in quantifying the gait impairment in stroke.

Another important aspect of our study was the task-specific approach [58,59] since the gait assessments were conducted in an outdoor environment. Our findings suggested that severely impaired stroke subjects displayed more variable gait smoothness during the 10 MWT trial. Although we did not control for intervening factors, such as the number of people or climate/soil conditions, this may indicate that it is difficult for more impaired subjects to deal with real-life constraints to maintaining a smooth gait [58]. These smoothness changes during walking may constitute relevant functional consequences and should be explored in future studies using a similar ecological approach. The use of IMUs allowed researchers to quantify gait in real-life situations, and the use of smoothness measures from IMUs allows the gait assessment in different ways. Since IMU sensors provide abundant information, future studies may also implement an approach using wearable sensors and machine learning techniques to detect subtle gait alterations of stroke survivors, similar to PD [60].

This study has some limitations to be considered, for example, the low sample size of the subgroup analysis. Further research should address the subtle smoothness changes in different levels of post-stroke impairments. Nonetheless, as mentioned above, the results indicated that severe stroke participants displayed greater variability of movement smoothness along the 10 meters of outdoor walking than controls and mild stroke participants. To the best of our knowledge, the present study is the first to evaluate the gait smoothness of post-stroke individuals walking in a "real context"–i.e. mimicking functional activity challenges during everyday life [41]. Another limitation is the sample heterogeneity, particularly in age and time post-stroke. Nonetheless, we found no relationship between these factors and SPARC. Further studies may analyze the relationship between SPARC and data acquired using scales to assess balance (such as the BERG balance scale) [39] and other functional walking tests (such as the Functional Ambulance Categories [61]). Finally, it is still necessary to investigate if gait smoothness is related to other clinical aspects, such as the risk of falling.

This study demonstrated that the SPARC analysis can be applied to quantify gait smoothness in post-stroke individuals in an outdoor environment. Our results showed that chronic post-stroke participants presented lower SPARC values (i.e. reduced smoothness) than controls. The convergence of results suggests that the reduced gait smoothness saw post-stroke might be due to impaired mediolateral trunk movements with respectively increased instability, as previously described [62]. The independence of SPARC from gait speed and spatiotemporal symmetry suggests that movement smoothness may add information to the traditional gait speed assessment of the 10 MWT. In this line of thought, it is reasonable to think that a rehabilitation program may focus on increasing gait speed and maintaining or reducing unsmooth movements to prevent falls. Nonetheless, further studies are needed to investigate the relationship between gait smoothness, gait symmetry, and risk of falls. In brief, smoothness evaluation adds information about movement quality, and it could be used as an additional measure to help clinicians in quantifying results of gait rehabilitation in post-stroke subjects.

## Supporting information

S1 Table Correlations between clinical parameters and Jerk values in the stroke and control groups. (TIF)

S1 File (XLSX)
