# Supplementary figures and images for: Movement smoothness in chronic post-stroke individuals walking in an outdoor environment—A cross-sectional study using IMU sensors
Source: PLoS One. 2021 Apr 22;16(4):e0250100. doi: 10.1371/journal.pone.0250100 (PMC8061986; doi:10.1371/journal.pone.0250100)

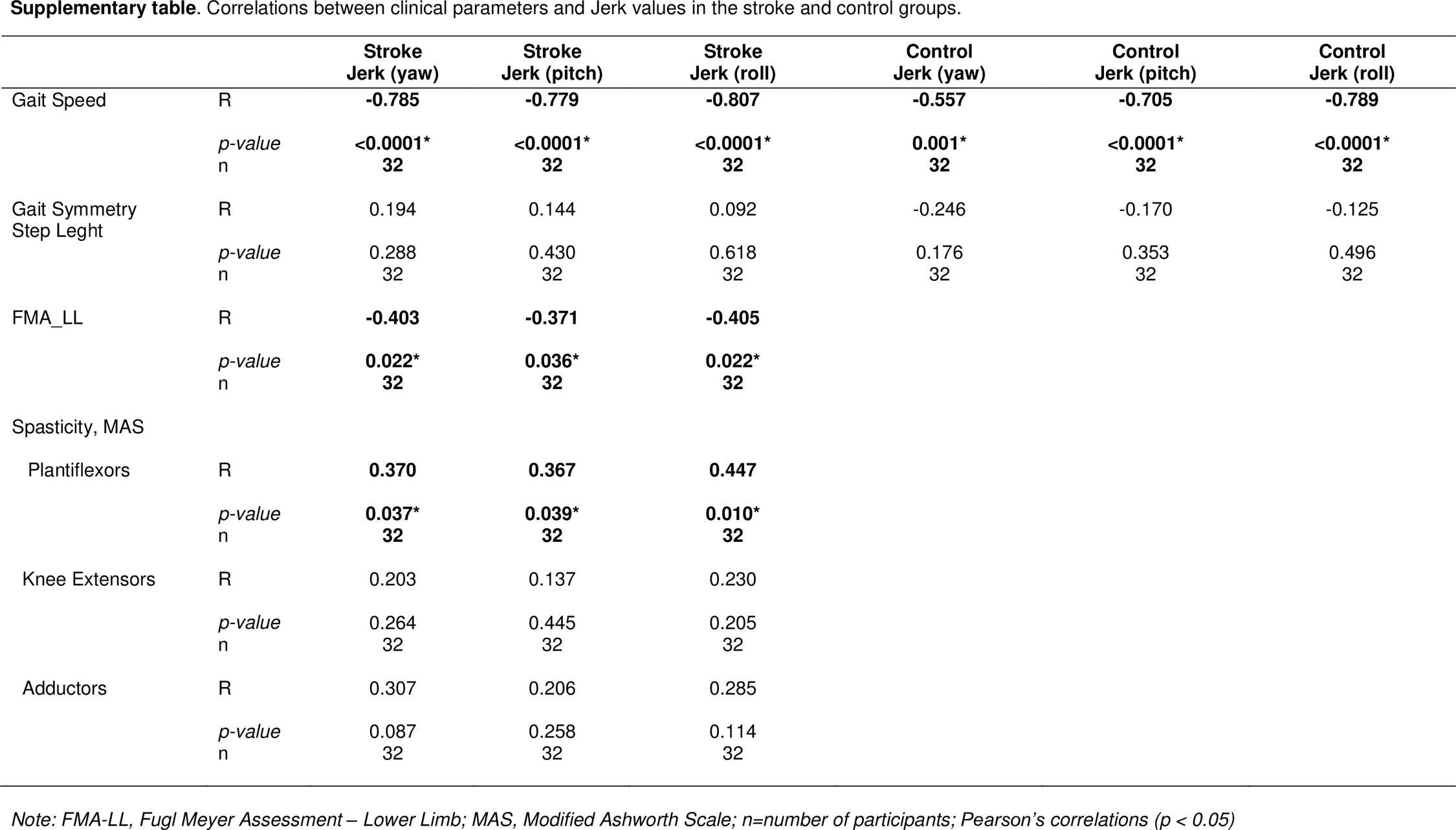

Supplement: S1 Table — (TIF) [file pone.0250100.s001.tif]
